# Urogenital microbiota-driven virulence factor genes associated with recurrent urinary tract infection

**DOI:** 10.3389/fmicb.2024.1344716

**Published:** 2024-02-07

**Authors:** Lei Jiang, Haiyun Wang, Lei Luo, Xiangyu Pang, Tongpeng Liu, Lijiang Sun, Guiming Zhang

**Affiliations:** Department of Urology, Affiliated Hospital of Qingdao University, Qingdao, China

**Keywords:** recurrent urinary tract infection, urobiome, virulence factor genes, non-invasive diagnostic, metagenomics, urine

## Abstract

Urinary tract infections (UTIs) are a common health issue affecting individuals worldwide. Recurrent urinary tract infections (rUTI) pose a significant clinical challenge, with limited understanding of the underlying mechanisms. Recent research suggests that the urobiome, the microbial community residing in the urinary tract, may play a crucial role in the development and recurrence of urinary tract infections. However, the specific virulence factor genes (VFGs) driven by urobiome contributing to infection recurrence remain poorly understood. Our study aimed to investigate the relationship between urobiome driven VFGs and recurrent urinary tract infections. By analyzing the VFGs composition of the urinary microbiome in patients with rUTI compared to a control group, we found higher alpha diversity in rUTI patients compared with healthy control. And then, we sought to identify specific VFGs features associated with infection recurrence. Specifically, we observed an increased abundance of certain VGFs in the recurrent infection group. We also associated VFGs and clinical data. We then developed a diagnostic model based on the levels of these VFGs using random forest and support vector machine analysis to distinguish healthy control and rUIT, rUTI relapse and rUTI remission. The diagnostic accuracy of the model was assessed using receiver operating characteristic curve analysis, and the area under the ROC curve were 0.83 and 0.75. These findings provide valuable insights into the complex interplay between the VFGs of urobiome and recurrent urinary tract infections, highlighting potential targets for therapeutic interventions to prevent infection recurrence.

## Introduction

Urinary tract infections (UTI) pose a significant burden on individuals and healthcare systems worldwide (Jhang and Kuo, 2017; Gaitonde et al., 2019). The UTIs are one of the most common bacterial infections, affecting millions of people each year. While UTIs can occur in both males and females, women are more commonly affected due to anatomical and hormonal factors (Jhang and Kuo, 2017; Gaitonde et al., 2019). Recurrent UTIs (rUTI) are defined as the occurrence of at least two symptomatic infections within a 6 months period or three infections within a year (Malik et al., 2018; Anger et al., 2019). This recurrent nature of UTIs not only leads to prolonged discomfort for patients but also contributes to antibiotic overuse and the development of antibiotic resistance (Neugent et al., 2022). Unfortunately, the precise mechanisms underlying the rUTI remain poorly understood.

Emerging evidence suggests that the urobiome, comprised of a diverse community of microorganisms inhabiting the urinary tract, may play a critical role in the development and recurrence of urinary tract infections (Wolfe and Brubaker, 2019). The urobiome is not simply a sterile environment but rather a complex ecosystem that can influence the host immune response and disease susceptibility (Karstens et al., 2016; Bučević Popović et al., 2018). Disturbances in the composition and functionality of the urobiome have been associated with UTIs and may contribute to infection recurrence (Neugent et al., 2022). Understanding the underlying factors driving recurrent urinary tract infections is essential for developing effective prevention and treatment strategies. Investigating the relationship between the urobiome and infection recurrence may provide valuable insights into novel therapeutic targets and approaches to manage recurrent urinary tract infections.

In recent years, there has been growing interest in elucidating the precise mechanisms through which microorganisms exert pathogenic effects (Liu et al., 2022; Rudin et al., 2023). One such mechanism involves the presence of virulence factors genes (VFGs), which are encoded by the microbial genome (Colbert et al., 2023). VFGs are molecular components or proteins produced by microorganisms to enhance their capacity to colonize and infect host tissues (Liu et al., 2022). However, effectively utilizing VFGs to assess the virulence characteristics of the microbiome and employing them for disease diagnosis presents certain feasibility challenges.

In this study, we aimed to assess the contribution of VFGs in urobiome to UTI and rUTI. We identified characteristic VFGs associated with UTI and rUTI. These VFGs can provide valuable insights into our understanding of rUTI and aid in the diagnosis of rUTI.

## Materials and methods

### Shotgun metagenomic sequence collection and quality control

**Figure 1:**
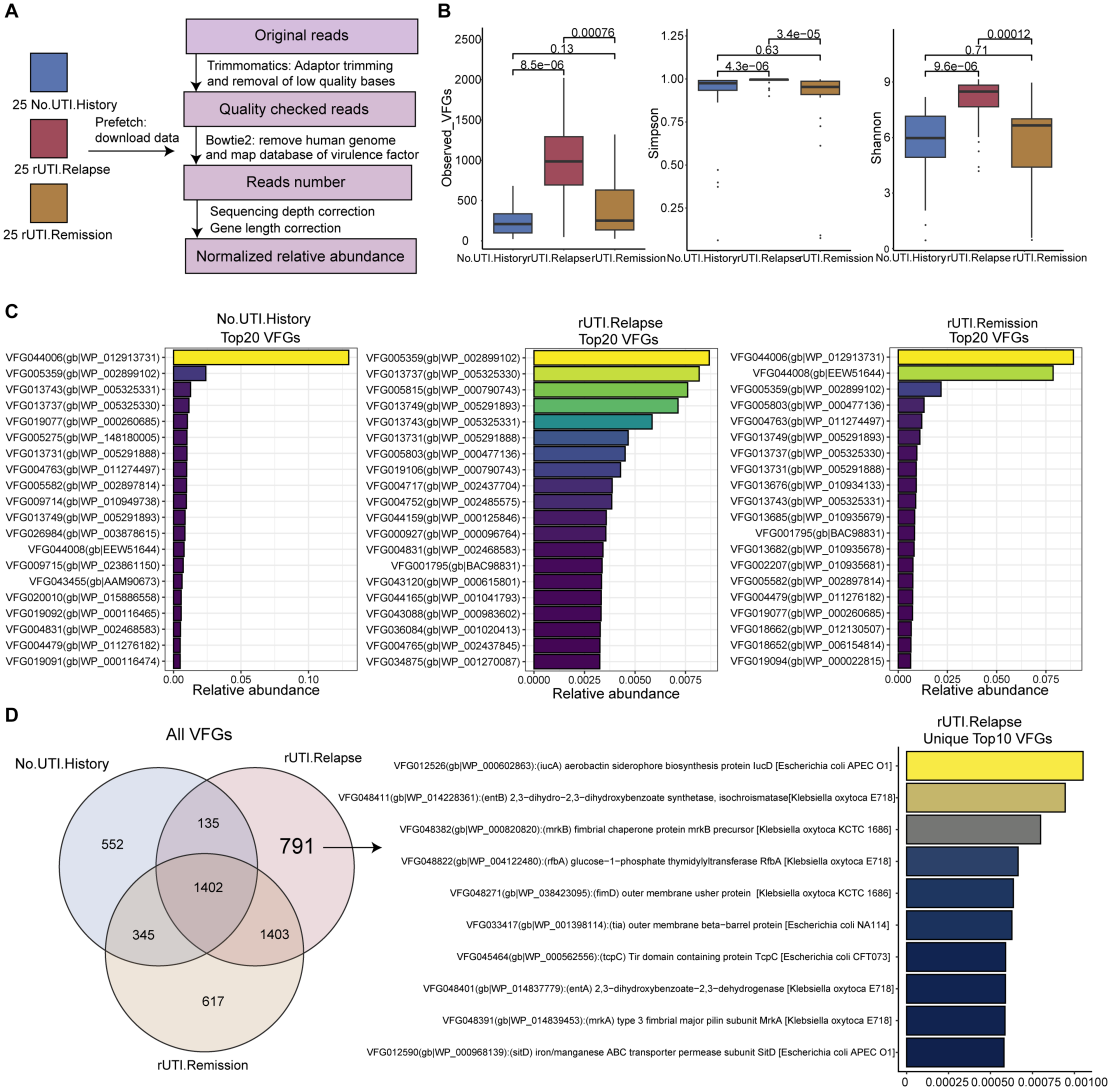
Workflow of this study and virulence factor diversity of microbiome. **(A)** Workflow of this study. **(B)** Alpha diversity of VFGs, including the number of observed VFGs, Simpson and Shannon diversity index. **(C)** The top 20 VFGs among three groups. **(D)** The overlap of VFGs among three groups.

In the Neugent et al. (2022) study, a total of 50 urine samples were collected from patients divided into two groups: 25 with UTI relapse and 25 in remission. Additionally, 25 urine samples were obtained from healthy individuals as controls. All samples in this study were from female participants. The study participants had no underlying issues affecting their urinary tract, immune system, and did not use indwelling or intermittent catheters. To retrieve the required data, we utilized the prefetch v2.10.7 tool from the National Center for Biotechnology Information (NCBI), enabling us to download the necessary datasets for our analysis. Figure 1A outlined the process, providing a visual representation of the entire workflow involving data collection and subsequent processing steps.

To ensure the sequencing data’s quality, we utilized Trimmomatic v0.39 (Bolger et al., 2014) to remove adapter sequences and low-quality bases. The parameters used were as follows: ILLUMINACLIP:TruSeq3-PE-2.fa:2:30:10:8:true TRAILING:20 MINLEN:60. Following quality control, an additional processing step was employed to eliminate human genomic sequences. For this purpose, we employed bowtie v2.4.4 (Langmead and Salzberg, 2012) and used the T2T-mY-rCRS genome (Nurk et al., 2022), which can be found at https://github.com/marbl/CHM13. This genome includes hard-masked PARs on chrY replaced with “N” and mitochondrion replaced with rCRS. This approach effectively removed any human genomic contamination.

### VFGs annotation

After removing human genomic sequences, we proceeded to align the remaining reads against the virulence factor database (VFDB) (Liu et al., 2022). This database, which can be accessed at http://www.mgc.ac.cn/VFs/main.htm, contains a comprehensive collection of virulence factors. For the alignment process, we utilized bowtie v2.4.4 ^13^ and performed subsequent analysis using samtools v1.13 (Li et al., 2009). By aligning the reads against the VFDB_setB_nt database (available at http://www.mgc.ac.cn/VFs/download.htm), we were able to identify the number of reads corresponding to VFs in each sample. Subsequently, for any sample *N*, we calculated the abundance as follows (Equations (1) and (2)), (Ma et al., 2020; Chang et al., 2021):

Step 1: Calculation of the copy number of each gene:


(1) $$b_{i}=\sqrt{\frac{x_{i}}{L_{i}}}$$

Step 2: Calculation of the relative abundance of gene *i*


(2) $$a_{i}=\sqrt{\frac{b_{i}}{\sum_{i}b_{i}}}$$

*a*_i_: the relative abundance of gene *i*, *b*_i_: the copy number of gene *i* from sample *N*, *L*_i_: the length of gene *i*, *x*_i_: the number of mapped reads.

This valuable information will be utilized in subsequent analyses to gain a comprehensive understanding of the involvement of oral microbiome-encoded VFGs in rUTI.

### Diagnostic model construction

To develop a diagnostic model for rUTI, we adopted a two-tiered strategy, utilizing both support vector machine (SVM) (Huang et al., 2018) and random forest (RF) classifiers (Rigatti, 2017). Initially, we trained a suite of standard classifiers: random forest, gradient boosting, SVM, and logistic regression. These classifiers were trained with default parameters to establish a baseline performance. Their accuracy was subsequently assessed on test data, with results collated in a dictionary for straightforward comparison. The SVM classifier demonstrated superior accuracy in differentiating between “No UTI History” and the combined “rUTI Relapse” and “rUTI Remission” categories, guiding our choice of SVM for the binary classification task.

#### SVM for binary classification

We first categorized the data into two groups: “No UTI History” and a combined category of “rUTI Relapse” and “rUTI Remission.” This classification was pivotal for distinguishing between individuals without a UTI history and those with rUTI, regardless of their current status. After data preprocessing and partitioning into training and test sets, we applied the SVM classifier, fine-tuning it with parameters *C* = 7, gamma = 600, and kernel = “rbf.” To optimize our SVM model, we executed a grid search to pinpoint the best hyperparameters.

#### RF for rUTI relapse vs. remission

In the model’s second tier, we concentrated on the “rUTI Relapse” and “rUTI Remission” samples, aiming to discern between relapse and remission phases. Employing the RF classifier, we adjusted the model with parameters max_depth = 61 and n_estimators = 230.

### Statistical analysis

The statistical analyses in this study were conducted using RStudio. We used the vegan package to calculate alpha diversity measures, such as the Shannon and Simpson indices. The Bray distance was directly computed on the VFG profiles. For principal coordinate analysis (PCoA), we utilized the ade4 package in R (Zapala and Schork, 2006). To assess the significance of group differences, we performed Adonis analysis using the vegan package. To test for differential abundances of VFGs, we employed the Kruskal rank-sum test. The *p*-values were adjusted using the Benjamini–Hochberg (BH) procedure, with a significance threshold set at a *p*-adjust value of <0.05. We used the ggplot2 package to create boxplots and PCoA plots. The pheatmap package was utilized to construct heatmaps visualizing the patterns of VFG abundances. The ggtern was used for the plotting of ternary diagrams (Hamilton and Ferry, 2018). Furthermore, we utilized the pROC package to generate ROC curves, which were employed to evaluate the performance of diagnostic models.

## Results

### Alpha difference associated with rUTI

According to the workflow depicted in Figure 1A, we obtained the relative abundance of VFGs in all samples. Subsequently, we calculated the alpha diversity measures of the three groups, including observed VFGs, Simpson, and Shannon indices based on the abundance of VFGs. Consistent results were observed, indicating that the alpha diversity of VFGs in the urobiome of patients with rUTI was significantly higher compared to those without a history of UTI (Figure 1B, P-values, observed VFGs: 8.5e−6, Simpson: 4.3e−6, Shannon: 9.6e−6). Furthermore, the alpha diversity was significantly higher in patients with recurrent UTI compared to those with a history of UTI but without recurrence (*p*-values, observed VFGs: 0.00076, Simpson: 3.4e−5, Shannon: 0.00012). There were no notable differences between individuals without a history of UTI and those without recurrence. These results suggest that VFGs may contribute to the recurrence of UTI.

### Core and unique VFGs in three group

First, we presented the top 20 abundant VFGs (Figure 1C and Supplementary TableS1) in each group, which we considered as core VFGs. We found that VFGs in urine mainly originated from bacterial genera such as *Gardnerella*, *Streptococcus*, *Corynebacterium*, and *Staphylococcus*. However, patients with rUTI had more virulence factors associated with *Escherichia coli* O157:H7. Furthermore, we identified unique VFGs in each group, including 552, 671, and 791 unique VFGs in the groups without history of UTI, non-recurrent UTI, and rUTI, respectively (Figure 1D). We presented the top 10 features in rUTI, which were distinctly associated with two typical pathogens, *Klebsiella* and *E. coli* O157:H7. This finding once again highlights the presence of unique VFGs in the urine of rUTI patients.

### Beta difference among three group

**Figure 2:**
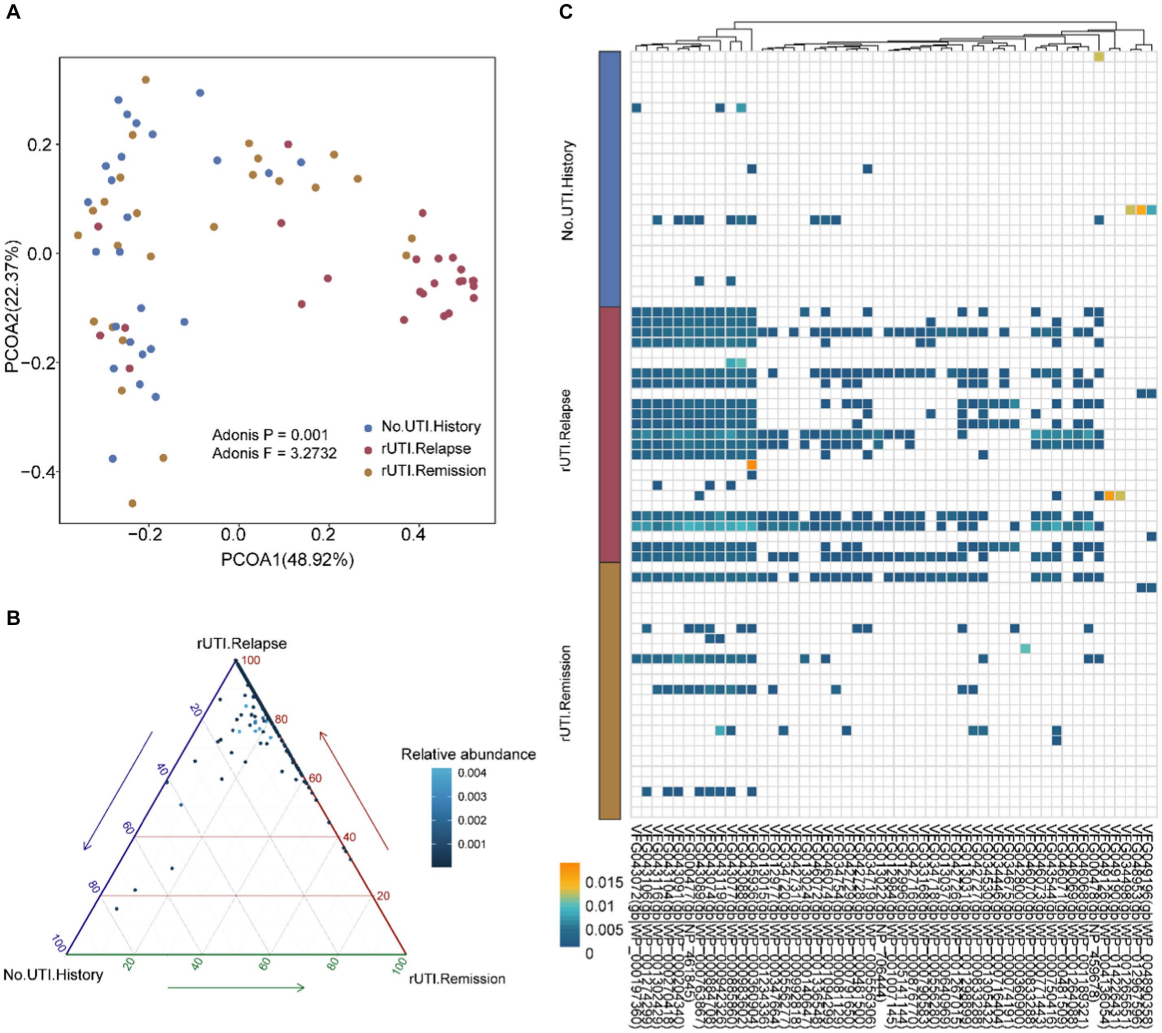
Beta diversity and differential VFGs. **(A)** Beta diversity based on bray-cutis distance and differential VFGs profiles. **(B)** Different VFGs with the *p*-values were adjusted using the BH procedure, with a significance threshold set at a p-adjust value of <0.05. **(C)** Top 50 different VFGs were showed using heatmap.

In order to further determine if there are compositional differences in VFGs among the three groups, we conducted PCoA analysis. Based on the Adonis test, we found significant differences in the composition of VFGs from a beta diversity perspective (Figure 2A, *p* = 0.001, *F* = 3.2732). To identify these differential VFGs more explicitly, we represented them using ternary plots with adjusted *p*-values below 0.05 (Figure 2B). We discovered that the majority of differentially abundant VFGs were enriched in women with rUTI, strongly suggesting their potential contribution to UTI recurrence. Additionally, we presented the top 50 VFGs (Figure 2C and Supplementary Table S2), such as VFG043077(gb|WP_000885860). VFG043119(gb|WP_000942326) and VFG043088(gb|WP_000983602). We found that these VFGs are primarily associated with *E. coli* O157:H7 (Supplementary Table S2). It is evident that individuals with a history of UTI and those prone to recurrence exhibit distinct VFG characteristics.

### Associations between VFGs and clinical data

**Figure 3:**
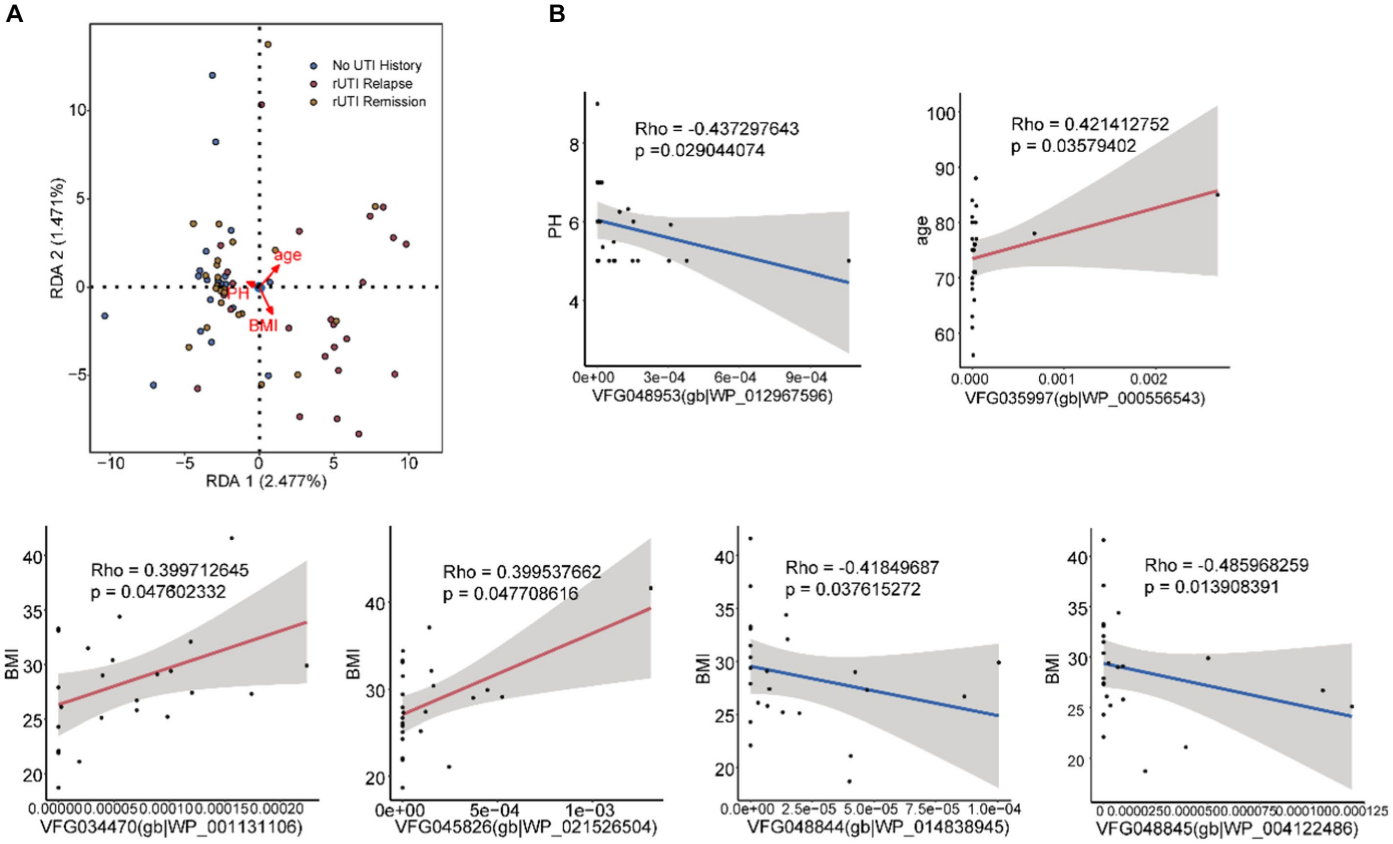
The associations between VFGs and clinical data. **(A)** Redundancy analysis. **(B)** Clinical data, urine pH, age, BMI.

To further elucidate potential mechanisms, we investigated the associations between VFGs abundance and clinical data, including age, BMI, and urine pH. We identified six significant correlations using Spearman correlation analysis and canonical correspondence analysis (Figures 3A,B and Supplementary Table S3). One correlation was found with urine pH (Figure 3B and Supplementary Table S3, VFG048953(gb|WP_012967596)), one with age (Figure 3B and Supplementary Table S3, VFG035997(gb|WP_000556543)), and the remaining four with BMI (Figure 3B and Supplementary Table S3, VFG034470(gb|WP_001131106), VFG045826(gb|WP_021526504), VFG048844(gb|WP_014838945) and VFG048845(gb|WP_004122486)).

### The rUTI diagnosis model based VFGs

**Figure 4:**
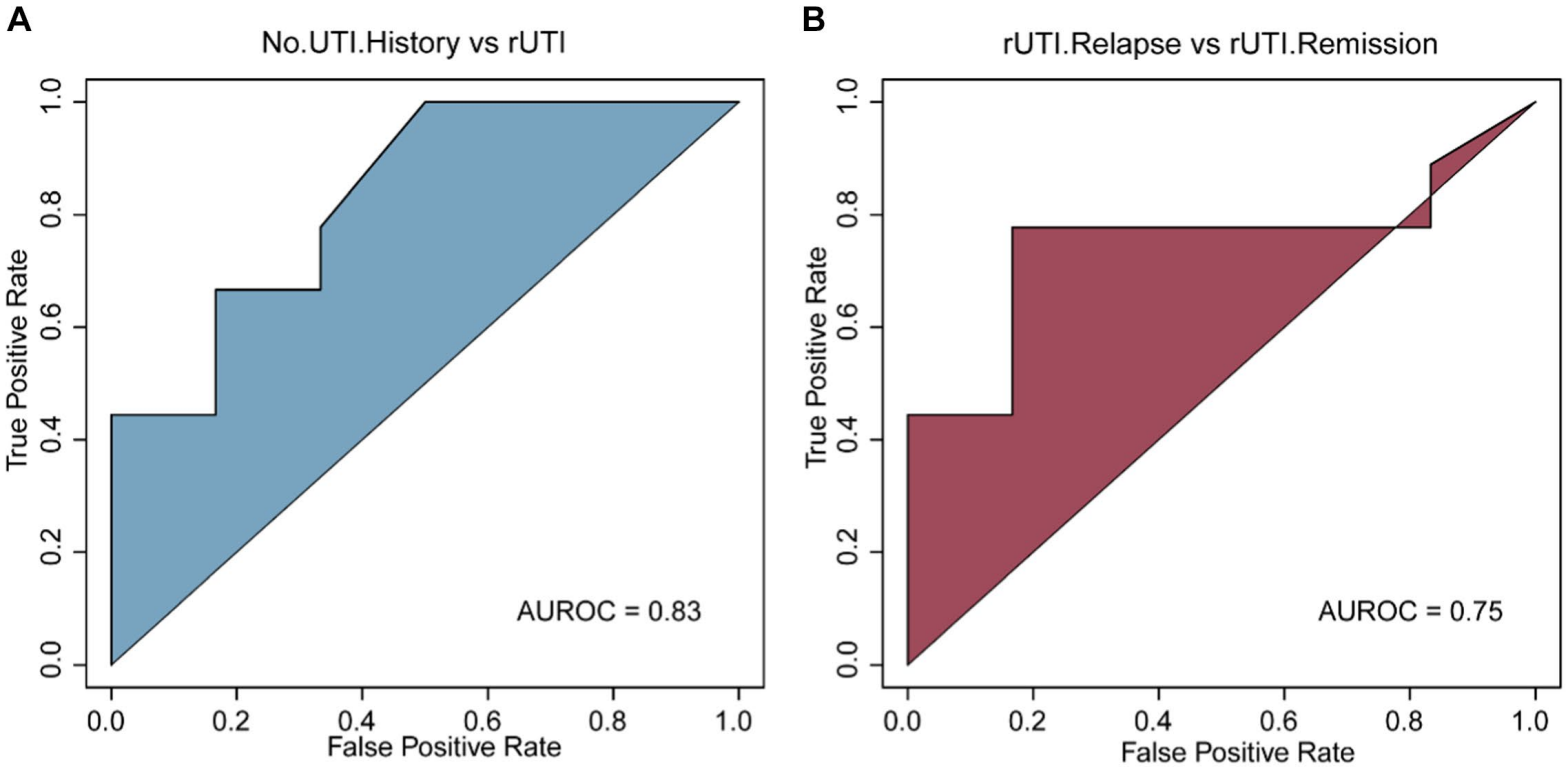
Construction of the diagnostic model. **(A)** No UTI history vs. rUTI based on SVM model. **(B)** rUTI relapse vs. rUTI remission based on RF model.

Further investigation is warranted to determine the potential of VFGs profiles in identifying patients with a history of UTI. We developed a machine learning model utilizing SVM to distinguish between females without a UTI history and those with a UTI history. Our findings suggest that the model can achieve an accuracy of 83% (Figure 4A). Moreover, our objective is to create a predictive model that utilizes urobiome information, specifically VFGs, to anticipate UTI recurrence. To accomplish this, we employed a random forest model to evaluate the diagnostic performance of VFGs in detecting rUTI, yielding an accuracy of 75% (Figure 4B). Overall, VFGs at the level of urobiome hold promise as indicators for both UTI history and recurrence. Our combined application of SVM and RF classifiers facilitated the creation of a comprehensive rUTI diagnostic model. The SVM classifier adeptly categorized individuals based on UTI history, while the RF classifier further refined the diagnosis by differentiating relapse and remission states. This dual approach ensures a thorough and precise diagnosis, setting the stage for tailored therapeutic interventions. Future endeavors might incorporate additional features or investigate alternative machine learning algorithms to augment the model’s precision.

## Discussion

UTI is a frequent and burdensome health issue that affects millions of people worldwide (Gaitonde et al., 2019). While UTIs can generally be treated with antibiotics, some individuals experience rUTI, which present a clinical challenge due to their frequent relapses and limited understanding of the underlying mechanisms. Recent studies have begun to explore the role of the urobiome. The urobiome is believed to contribute to the maintenance of urinary tract health by promoting a balanced microbial environment and resistome (Neugent et al., 2022). However, the annotation of VFGs may provide more direct insights into the microbial contributions to the recurrence of urinary tract infections.

In our study, these VFGs were predominantly associated with two typical pathogens, *Klebsiella* and *E. coli*, further reinforcing their role in rUTI. *E. coli* has been reported to be enriched in rUTI patients and extremely rare in healthy controls and non-recurrent individuals (Magruder et al., 2019; Tornic et al., 2020; Neugent et al., 2022; Worby et al., 2022) and urinary pathogenic *E. coli* is the primary cause of UTI (Lo et al., 2017). which is consistent with our annotation of more *E. coli*-associated VFGs. Previous study revealed that a specific adaptation of *E. coli* to the bladder environment would be predicted to result in decreased fitness in other habitats, such as the gut (Chen et al., 2013). Hence, the virulence factors we are concerned with will provide critical evidence for how *E. coli* colonizes, invades, and acts in the urinary tract. Recent, Naboka et al. (2020) discovered that the Enterobacteria obtained from the urine of female patients experiencing rUTI exhibit a diverse range of VFGs based on qPCR, potentially contributing to the persistence of chronic inflammation in the lower urinary tract. We revealed consistent results using the metagenome.

Although there are many studies predicting urinary tract infections (Heckerling et al., 2007; Gadalla et al., 2019; Sanaee et al., 2020), few have focused on predicting recurrence of urinary tract infections (Cai et al., 2014). To address this, we developed a diagnostic model using machine learning algorithms, namely random forest and support vector machine analysis, based on the levels of the identified VFGs. Our combined application of SVM and RF classifiers facilitated the creation of a comprehensive rUTI diagnostic model, yielding a high accuracy. Our model can help with treatment decisions and enhance clinical outcomes.

Collectively, our findings contribute to the growing body of evidence implicating the role of the urobiome and VFGs in the recurrence of urinary tract infections. By identifying the specific VFGs associated with rUTI, our study provides potential targets for therapeutic interventions aimed at preventing infection recurrence. Future research is warranted to further elucidate the underlying mechanisms by which these VFGs contribute to the pathogenesis of rUTI. In conclusion, our study sheds light on the complex interplay between the VFGs of the urobiome and recurrent urinary tract infections. The identification of specific VFGs associated with rUTI paves the way for the development of personalized treatment strategies and diagnostic approaches, ultimately improving the management of recurrent urinary tract infections.

## Data availability statement

The original contributions presented in the study are included in the article/Supplementary material, further inquiries can be directed to the corresponding authors.

## Ethics statement

Ethical approval was not required for the study involving humans in accordance with the local legislation and institutional requirements. Written informed consent to participate in this study was not required from the participants or the participants’ legal guardians/next of kin in accordance with the national legislation and the institutional requirements.

## Author contributions

LJ: Validation, Visualization, Writing – original draft. HW: Software, Writing – original draft. LL: Conceptualization, Writing – original draft. XP: Data curation, Writing – original draft. TL: Methodology, Writing – original draft. LS: Project administration, Resources, Writing – review & editing. GZ: Supervision, Writing – review & editing.
